# Short video users’ personality traits and social sharing motivation

**DOI:** 10.3389/fpsyg.2022.1046735

**Published:** 2022-12-09

**Authors:** Zhang Da-yong, Su Zhan

**Affiliations:** ^1^State Key Laboratory of Communication Content Cognition, People's Daily Online, Beijing, China; ^2^Key Laboratory of Interactive Media Design and Equipment Services Innovation, Harbin Institute of Technology, Harbin, China

**Keywords:** short video, social sharing, motivation, personality traits, uses and gratifications theory

## Abstract

**Purpose/significance:**

Studying the correlation between short video users’ personality traits and their sharing motivation can enrich the theoretical research on social sharing motivation and provide a reference for short-video content management and platform construction.

**Methods/process:**

Based on uses and gratifications theory and personality traits theory, a structural model affecting short-video users’ sharing motivations was proposed. A total of 579 valid questionnaires were collected from a social network, and the proposed hypotheses were tested using SPSS and Amos software.

**Results/conclusion:**

The results show that the personality traits of short-video users affect their sharing motivation and that their specific sharing motivation also differ due to their personality traits. At the same time, the research results also confirm the Matthew effect of “the rich getting richer” and the social compensation effect of “the poor getting richer” in the context of social platforms that host short videos.

## Introduction

Given universal access to the internet and the wide popularity of smart terminals, various social applications have become important channels for people to meet their needs for information, entertainment and interpersonal communication. There are more than 3.8 billion social media users worldwide (Gupta et al., 2021). Many daily activities, such as sharing, communicating, commenting, liking, etc., take place on social media. According to relevant research, on average, users spend 2.4 h a day on social media (Shen et al., 2020). The 47th China Statistical Report on the Internet Development (CNNIC, 2021) shows that China’s internet penetration rate reached 70.4% in 2020 and that the number of internet users in the country reached 989 million; within this group, the number of short-video users was 873 million, accounting for 88.3% of all internet users. While short video use is increasing rapidly in China, short-video application platforms represented by Kuaishou and TikTok are also expanding rapidly in overseas markets, with global downloads of TikTok reaching 626 million in the first half of 2020, making it the most downloaded application in the world. The overseas version of Kuaishou launched during the same period performed outstandingly in Brazilian, South Korean, Russian and Southeast Asian markets, reaching an average of 481 million active users per month in 2020, a number that increased to 519.8 million in the first quarter of 2021; the app also experienced significant growth in the viscosity of active users.

Social sharing can direct large volumes of traffic to content providers and social media, which is key to determining the success or failure of social media. The question of how to motivate users to participate in content sharing and thus enhance the value of content providers and social media alike has become a hot topic in both industry and academia (Barbera et al., 2009). The current research focuses on user profiles to investigate ways of improving the recommendation accuracy of content products and thus enhancing user viscosity (Li et al., 2006). The process of constructing user profiles is essentially based on the collection and analysis of user data to develop a labelling system that can describe user characteristics and accurately model user needs (Teixeira et al., 2015). Personality is a stable and habitual way of thinking and style of behaviour that conveys an overall picture of the individual’s unique psychological characteristics; motivation, on the other hand, is the driving factor that inspires users to engage in specific behaviours. These two factors constitute the basis and foundation for constructing user profiles. However, to date, few studies have investigated the correlation between users’ personality traits and sharing motivations, and the intrinsic motivational factors that drive users’ participation in sharing require further exploration (Barrick, 2005). The study of short-video users’ sharing motivations can enrich the theoretical research concerning motivations for social sharing behaviour motivation and thus serve as a reference for short-video content management and platform construction.

## Literature review

### User personality traits in social networks

Personality is the dynamic organization of one’s internal psychophysical system that is reflected in one’s thoughts and behaviours and determines one’s unique behaviours and thoughts in response to the environment. Traits are neuropsychological structures that enable many stimuli to be equally effective in triggering certain behaviours and can create and guide a variety of behavioural expressions that are functionally equivalent and consistent. G.W. Allport, the founder of the trait theory of personality in modern psychology, claimed that personality is composed of various traits, that traits can be treated as persistent (exhibiting temporal continuity) and stable (exhibiting situational consistency) patterns of behaviour and that experiments can produce an understanding of individual personality traits and allow us to predict individual behavioural responses (Allport, 1943). Based on Allport’s research, R.B. Cattell introduced factor analysis into personality research, extracted 16 root traits from human behaviour, and developed the “16 personality factors test” method (Cattell, 1948). Since that time, as research has progressed, scholars have found that the five-factor model, including extroversion, conscientiousness, neuroticism, openness, and agreeableness, is widely representative and has been extensively validated across cultures (Mccrae and Costa, 1997) and times (Hampson and Goldberg, 2006).

Early research on the five-factor model and user behaviour focused only on a limited number of aspects, such as personality and interpersonal relationships, personality and purchase propensity, and personality and career choice, with extraversion and conscientiousness being the most frequently studied personality traits (Forret and Dougherty, 2001; Robles-Granda et al., 2020). Due to the development of online technologies, especially the emergence of social networks, interpersonal communication has changed. Compared to offline modes of face-to-face communication, online interactions lead to a wider range of interpersonal connections that are not limited to maintaining one’s original social relationships (Ellison et al., 2007). In addition, without the constraints of face-to-face conversations, users who engage in online interactions are better able to talk about or disclose deeply personal issues; therefore, online interactions generate more self-expression and self-presentation (Lee et al., 2014). According to Amichai-Hamburger, personality traits are a major factor influencing how people behave online, and without understanding the personalities of online users, it is impossible to understand how the internet operates in depth (Amichai-Hamburger and Vinitzky, 2010). The link between personality traits and the extent of internet use has since been demonstrated by many psychological experiments. For example, the study by Correa et al. showed that extraversion and openness to experience were positively associated with the frequency of social media use, whereas emotional stability was negatively associated with the frequency of social media use (Correa et al., 2010). Liu and Campbell (2017) found that highly conscientious users maintain strict control over their online behaviours and present themselves with caution. It was found that the personality trait that has the greatest effect on online information-seeking strategies is conscientiousness. Agreeableness and neuroticism are related to several dimensions of social media-specific beliefs (Uslu and Durak, 2022). In addition, users’ preferences for specific features of social networks vary with their personality traits. Openness to experience is the personality factor that is most likely to be associated with trying new modes of communication and seeking novel experiences (Butt and Phillips, 2008). Users with extroversion and openness to experience participate in significantly more groups on social networks and use social features more frequently than introverted users (Ross et al., 2009). Similarly, users with high levels of neuroticism use the message board function of social networks more often, whereas those who are low in neuroticism prefer to upload photos; this difference can be attributed to the fact that people with neuroticism-related traits are better at controlling textual content than visual content (Kuss and Griffiths, 2011). There are some studies on personality traits and inappropriate use of social media. It was demonstrated that agreeableness, openness to experience and conscientiousness are negatively related to Facebook addiction, while loneliness, narcissism, impulsivity and shyness are significantly correlated with Facebook addiction (Rajesh and Rangaiah, 2022).

Some studies have focused on how the interaction of personality traits and social media affects certain aspects of life and society. The analysis of nationally representative survey data from the United States indicated that the interaction effects of social media use and the Big Five personality traits determined the formation of attitudes towards political compromise (Choi and Shin, 2017). Astleitner pointed out that personality traits and social media experiences are linked to mental health in a variety of ways (Astleitner et al., 2022).

### User sharing motivation in social networks

Motivation is the psychological tendency or internal drive that inspires and sustains an organism’s action and leads it towards a certain goal (Kleinginna and Kleinginna, 1981; Schunk, 1990). Modern motivation theory suggests that complete motivation should include three aspects: intrinsic needs, external inducements, and self-regulation (Osterloh and Frey, 2000). Social influence theory suggests that online users’ sharing behaviours are influenced by external factors; specifically, factors such as group norms and social identity affect individuals’ social perceptions, social attitudes and social behaviours. Social exchange theory suggests that users participate in sharing to obtain certain benefits, including both tangible rewards and intangible gains such as a sense of accomplishment or spiritual rewards (Cropanzano and Mitchell, 2005). From the perspective of users’ utility increment brought by the increase of network product users, the network externality theory discusses the positive impact of product user scale on the adoption intention or behavior of social networks (Gao and Bai, 2014). Uses and gratifications (U&G) theory adopts a psychological perspective to study media audiences and argues that audiences’ media choices depend on the level of satisfaction that they can derive from media use (Katz et al., 1973). In particular, when audiences are faced with more media choices, the satisfaction of their social or psychological needs is key to using those media. The key point that distinguishes U&G theory from traditional theory is that the former shifts from the traditional study of media effects, which focuses on “what the media does to people,” to an analysis of media audiences that asks “what people do to the media.”

Early studies of media use motivation viewed users as recipients rather than creators of media content; thus, studies of user motivations focused on need satisfaction in terms of entertainment, time-wasting, information acquisition, emotional expression or emotional release, and interpersonal dialog (Ruggiero, 2000). Social media provides a broad platform for users to create more original content, and the process of media selection is driven by a strong need for personalization. Interaction and sharing have become the main functions of social media, and users’ abilities to obtain wider social recognition or external connections *via* interaction and sharing has become the main motivation for internet use (Park et al., 2009; Hofmann and Nadkarni, 2012). Motivations for internet use can be classified into internal and external motivations, in which context internal motivations involve the pleasure and internal satisfaction that users can enjoy due to sharing or delivering content, while external motivations involve the personal image enhancement and positive social impact that users can have when they share or deliver content. Falgoust categorized six sharing motivations on TikTok consistent with U&G theory analysis: entertainment, convenience and utility for widespread communication, increasing social interaction, finding social support, seeking and sharing information, and escaping from everyday life (Falgoust et al., 2022). Some scholars have also introduced motivational predictors, such as sex, belongingness, self-presentation, and personality traits, to U&G theory studies. For example, Hofmann and Nadkarni argued that satisfying the need for belongingness and self-presentation are motivations for the continued use of social network (Hofmann and Nadkarni, 2012) and that the influence of personality traits on sharing motivation is mostly focused on three dimensions: extraversion, conscientiousness and neuroticism (Błachnio et al., 2013).

Some researchers have discussed the motivation of information-sharing activities in social media by classifying information into various types: personal, sensational, political, casual, and experience. Finally, they conclude that users’ motivations in sharing information tend to be consistent for each type of information, that is, to share the impression of social media users on a matter (Ghaisani et al., 2017). Some studies focus on sharing motivation in specific scenarios. Zhang proposes four important tourist photosharing motivations (enjoyment, altruism, self-expression, and social relationship) based on the two dimensions of extrinsic-intrinsic and self-centred-community-related motivation (Zhang et al., 2022). Interestingly, another study showed that recognition and status-seeking were more important motivations for sharing travel photos for Chinese tourists than for Western users (Li, 2020). Moreover, perceived enjoyment is the most important motivation for travel experience sharing, and security and privacy reasons are the biggest inhibitors of sharing information (Oliveira et al., 2020). Among the motivations for information sharing in the advertising business, in addition to the motivations of pleasure, altruism and social interaction, the research also analyses the specific motivations for such situations, such as liking/helping the brand (Lee et al., 2019), self-brand identity (Anggraeni and Diandra, 2017), and brand engagement (Ilich and Hardey, 2018).

### Relationship between users’ personality traits and sharing motivation for short videos

Short videos have received increasing attention from users and researchers due to their short durations and the large amounts of information they convey, which fully match users’ fragmented consumption habits. Most studies have focused on analyzing social network users’ adoption behaviors and continued usage intentions in terms of two aspects: intrinsic factors and external environmental factors. However, few studies have focused on the motivations underlying users’ sharing on social media. This study takes short-video users as its research object, integrates U&G theory and personality trait theory, and proposes a structural model of short-video users’ sharing motivations, intending to highlight the intrinsic connection between personality traits and short-video users’ sharing motivations and explore a clearer and more comprehensive causal chain leading from personality to behavior research. The study also intends to explore the causal relationship between users’ personality traits and sharing motivations for short videos. This study is conducted to address the following three research questions. (1) How can the typical personality traits of short-video users be measured? (2) What are the social sharing motivations of short-video users? (3) What influence do the personality traits of short-video users have on their sharing motivations?

To answer the first question, in terms of personality trait selection, this study explores the associations among the three personality traits of conscientiousness, extraversion and neuroticism, which exhibit significant differentiation concerning users’ motivations to share. Social media users’ motivations to use is not a single variable, and users’ engagement in sharing behaviours is reflected not only in the varying degrees of engagement motivation among individuals but also in their types of motivation for engagement. Some users are influenced in their sharing decisions by intrinsic motivations such as their interests, curiosity, and values, while others are influenced by their external environments or groups. To address the second question, studies have shown that first, the pleasure and satisfaction derived from contributing knowledge to help others can be a form of intrinsic motivation and that reciprocity can lead to intangible rewards (e.g., online reputation or influence), which can further motivate users to participate in information sharing (Wei, 2009); second, releasing emotions, clarifying positions or opinions, correcting self-judgements, and gaining social recognition are the main motivations for users to participate in such groups (Berger and Buechel, 2012); third, active social engagement enhances the user’s recognition in the group, which in turn enhances their online presence (Błachnio et al., 2013); and fourth, research based on uses and satisfaction theory suggests that media, especially social media, is used to pass the time rather than to satisfy a specific need (Ruggiero, 2000). Both content generators and social media cultivate the usage habits of users *via* content creation and the development of an online ecology to produce a stable user base. Based on the comprehensive consideration of existing research results and the characteristics exhibited by users’ sharing behaviours, this study summarizes short-video users’ sharing motives in terms of four aspects: image management, altruism, emotional expression and pastime and conformity. Concerning the third issue, this study conducts hypothesis testing as follows.

Among the Big Five personality traits, conscientiousness is used to describe a personality’s degree of control, self-management and effort in task performance, such as in terms of a sense of mission or the responsibility to accomplish certain tasks and goals. Responsible people control their impulses, are organized, self-disciplined, cautious, diligent, and planning-focused, and make great efforts to achieve their goals (Ashton and Lee, 2001), while people who lack responsibility tend to act on impulse, procrastinate and be unorganized. It has been shown that people who are not responsible use social networks more frequently and are more likely to become addicted to them (Ross et al., 2009); in contrast, highly conscientious users have significantly more friends but share less information online (Karl et al., 2010) because they may perceive that the time they spend on social media distracts them from pursuing their own goals and reduces their efforts to achieve those goals. In addition, research has suggested that highly conscientious users are less likely to exhibit emotional problems associated with social media use (Glass et al., 2014). In summary, the intensity of conscientiousness is inversely related to motivations such as image management, altruism, emotional expression, and pastime and conformity; i.e., the more responsible an individual is, the weaker his or her motivation for short-video sharing. Therefore, it is proposed that conscientiousness negatively affects users’ social sharing motivations, as expressed in the following hypotheses:

*H*1-a: Conscientiousness negatively influences users' motivation for image management.

*H*1-b: Conscientiousness negatively affects users' motivation for altruism.

*H*1-c: Conscientiousness negatively affects users' motivation for emotional expression.

*H*1-d: Conscientiousness negatively influences users' motivation for pastime and conformity.

Among the Big Five personality traits, extroversion represents the degree to which a person is social and outgoing. Extroverts tend to approach people and things energetically, enjoy contact with people, are friendly, talkative, confident, and energetic, seek social interaction, and enable those with whom they interact to feel positive emotions (Amichai-Hamburger et al., 2002); they also stimulate and direct interactive activities among members of the group (Lemoine et al., 2016). Introverts may be described as withdrawn and inactive, and they express fewer positive emotions. Two contrasting perspectives on the relationship between high and low scores on extroverted personality traits and social media use have emerged. On the one hand, some research has suggested that extroverts are expected to spend more time on social media because it creates a platform on which they can interact with their friends (Barbera et al., 2009). On the other hand, introverts may prefer to use social media to communicate, which can compensate for their lack of offline interpersonal skills (Muscanell and Guadagno, 2012) In addition, research has also shown that extroverts are more likely to join online groups and have a higher number of friends on the internet than introverts (Nicole and Winter, 2008). Therefore, it is proposed that extroversion as a personality trait positively influences-users’ motivation to share socially, as hypothesized below:

*H*2-a: Extroversion positively influences users' motivations for image management.

*H*2-b: Extroversion positively influences users' motivations for altruism.

*H*2-c: Extroversion positively influences users' motivations for emotional expression.

*H*2-d: Extroversion positively influences users' motivations for pastime and conformity.

Neuroticism is the antonym of emotional stability. Individuals with high levels of neuroticism are emotionally unstable and encounter difficulty coping with stress. They often feel anxious, angry and sad and prefer to be alone in real life rather than to interact with other people (John et al., 2008). It has been reported that users with neurotic traits generally use message board features that allow them to receive and post comments, while users with low neuroticism prefer to upload photos (Ross et al., 2009). This difference can be attributed to the fact that people with neuroticism-related traits are better at controlling textual content than visual content. Individuals with neuroticism-related traits present as anxious and sensitive and are less likely to display their original message in a virtual environment. However, other studies have obtained opposite findings, indicating that people who score high on neuroticism are more likely to post photos to their home pages (Amichai-Hamburger and Vinitzky, 2010). In addition, some studies have suggested that people who score high on neuroticism may be more inclined to spend time on social networks because using the internet allows them to avoid the discomfort of face-to-face communication, and they try to look as attractive as possible and may therefore spend longer thinking about what to say or how to disguise themselves (Ryan and Xenos, 2011; Muscanell and Guadagno, 2012). Overall, the findings concerning neuroticism suggest that people who score high on this trait tend to reveal information because they seek confidence online, while those who score low are emotionally stable and tend to express their thoughts by sharing information. In summary, the following hypotheses are proposed:

*H*3-a: Neuroticism positively influences users’ motivation for image management.

*H*3-b: Neuroticism positively influences users’ motivation for altruism.

*H*3-c: Neuroticism positively influences users’ motivation for emotional expression.

*H*3-d: Neuroticism positively influences users’ motivation for pastime and conformity.

After analysis and verification of the above hypotheses, we can further explore the Matthew effect and the social compensation effect in the context of social platforms that host short videos. The Matthew effect was originally used to explain a social phenomenon: “For to him who has shall be given and he shall have abundance; but from him who does not have, even that which he has shall be taken away” (Tang et al., 2000, p. 687). The term was later borrowed by economists to reflect the inequality of income distribution in winner-takes-all economics. The Matthew effect can powerfully explain and demonstrate many phenomena in the medical, economic and social fields (Fernández-Villaverde et al., 2021; Peters and Roose, 2022; Zhou et al., 2022). The compensation effect is an economic concept that holds that money needs to be compensated to maintain the original utility after the price increases. Gradually, the compensation effect has been applied to various fields of sociology. The social compensation effect is the mechanism by which the subject of an unbalanced allocation of interests can receive compensation in the face of social unfairness. In general, the Matthew effect increases inequality, while the social compensation effect reduces inequality.

## Materials and methods

### Measure

In this paper, an online questionnaire is used to investigate user personality traits and motivation for sharing short videos. The measure ask participants to imagine the short video they recently see online, rather than requiring them to watch a specific short video live. To ensure the quality of the questionnaire, this paper draws on the research of Karim et al. (2009) and Kim and Chung (2014) to select three typical personality traits, i.e., extroversion, conscientiousness and neuroticism (see Table 1), which are scored on a five-point Likert scale that includes five options for each variable ranging from “strongly disagree” to “strongly agree.” To investigate sharing motivation, an 18-question measure of the sharing motivation of short-video users was developed by reference to four aspects: image management, altruism, emotional expression and pastime and conformity (see Table 2); this measure also uses a five-point Likert scale and references the studies conducted by Syn and Oh (2015), Zhang et al. (2017) and Moghavvemi et al. (2018).

**Table 1:** User personality traits scale.

Variable	Number	Measurement issues	Source (of information etc.)
Extroversion	EX1	I think I can liven up the environment I’m in.	Karim et al. (2009)
	EX2	I feel relaxed when socializing with others	
	EX3	I talk to people all the time.	
	EX4	I do not mind being the centre of attention.	
Conscientious-ness	CO 1	I usually do things with thorough preparation.	Karim et al. (2009)
	CO 2	I’m very detail-oriented.	
	CO 3	I like to make plans.	
Neuroticism	NE 1	I always worry too much.	Kim and Chung (2014)
	NE 2	I get angry easily.	
	NE 3	I’m often emotionally unstable.	
	NE 4	I get depressed easily.	

**Table 2:** User motivation to share measurement scale.

Variable	Number	Measurement issues	Source (of information etc.)
Image management	XX1	Sharing that video will help me shape the image I want to build on social media.	Syn and Oh (2015)
	XX2	Sharing that video will allow others to get to know me better.	
	XX3	Sharing that video will help me show my personality.	
	XX4	I want to get recognition and positive comments from others by sharing that video.	
	XX5	I want to increase my reach by sharing that video.	
	XX6	I want to strengthen my connections with others by sharing that video.	
	XX7	I want to share that video to strengthen connections with like-minded people with similar interests.	
Altruism	LT1	I thought it might be useful to others to share the video.	Zhang et al. (2017), (Moghavvemi et al., 2018)
	LT2	I thought the video was interesting/useful, so I shared it with my circle.	
	LT3	I take the initiative to share interesting/useful short videos in the hope that others will share interesting/useful short videos with me in the future.	
Emotional expression	QG1	I wanted to confirm my judgement by sharing that video.	Syn and Oh (2015)
	QG2	I would like to express my attitudes and opinions by sharing that video.	
	QG3	I want to influence the attitudes and opinions of others by sharing that video.	
	QG4	I would like to gain social support by sharing that video.	
Pastime and conformity	XQ1	That video is shared for fun and entertainment and to pass the time.	Syn and Oh (2015)
	XQ2	Sharing videos is a habit of mine.	
	XQ3	I shared the video because it is popular to share videos in my social circle.	
	XQ4	I shared the video to receive certain rewards (such as special offers, etc.).	

### Procedure and participants

Ethical review and approval was not required for the study on human participants in accordance with the local legislation and institutional requirements. The participants provided their written informed consent to participate in this study. There were not specific invitations to the research included and any inclusion criteria. The research targeted people who use smartphones and like to access information on social platforms.

The questionnaires were distributed online *via* Wechat between March 1, 2021, and March 31, 2021; a total of 602 questionnaires were returned. The list deletion method was adopted to account for missing items, i.e., if one or more items were missing from each record of the questionnaire, the record was considered invalid and marked for deletion. A total of 579 valid questionnaires were obtained in this survey. The sample statistics are shown in Table 3. Among the participants, 281 were male, accounting for 48.53% of the total, while 298 were female, accounting for 51.47% of the total. The number of the respondents surveyed was equal across both genders. The study included 165 respondents aged 19 or below, accounting for 28.5% of the total; 317 respondents aged between 20 and 29, accounting for 54.75% of the total; 25 respondents aged between 30 and 39, accounting for 4.32% of the total; 43 respondents aged between 40 and 49, accounting for 7.43% of the total; and 29 respondents aged 50 or older, accounting for 5.01% of the total.

**Table 3:** Descriptive statistics and correlations.

Variables	Mean	SD	1	2	3	4	5	6	7	8	9	10
1. Age	25.56	10.36629										
2. Sex	1.51	0.5	0.090^*^									
3. Degree of Education	2.98	0.355	−0.011	−0.101^*^								
4. Participation Frequency	2.52	0.958	0.084^*^	0.047	0.024							
5. Conscientiousness	3.77	0.89627	0.167^**^	−0.074	0.124^**^	0.021						
6. Extroversion	3.43	0.93442	0.027	−0.175^**^	0.135^**^	0.066	0.730^**^					
7. Neuroticism	2.44	0.99615	−0.073	−0.064	0.017	−0.028	0.011	0.102^*^				
8. Image Management	2.82	1.06313	0.042	−0.147^**^	0.014	0.164^**^	0.352^**^	0.438^**^	0.252^**^			
9. Altruism	3.28	0.99608	0.120^**^	−0.089^*^	0.093^*^	0.192^**^	0.410^**^	0.451^**^	0.195^**^	0.727^**^		
10. Emotional Expression	2.83	1.07078	0.063	−0.182^**^	0.019	0.148^**^	0.335^**^	0.419^**^	0.289^**^	0.790^**^	0.718^**^	
11. Pastime and conformity	2.62	1.04415	−0.107^**^	−0.235^**^	0.123^**^	0.156^**^	0.251^**^	0.439^**^	0.367^**^	0.703^**^	0.567^**^	0.760^**^

*At 0.05 level (two-tailed), the correlations are significant. **At 0.01 level (two-tailed), the correlations are significant.

Structural equation model, a statistical method of analyzing the relationship between variables based on the covariance matrix of variables, was used as the analytical method in this research. Structural equation model has the advantages of processing multiple dependent variables at the same time, allowing independent variables and dependent variables to contain measurement errors, estimating factor structure and factor relationship at the same time, allowing greater elasticity of the measurement model, and estimating the fit degree of the whole model. Therefore, structural equation models are often used in confirmatory factor analysis, higher-order factor analysis, path and causality analysis, multi-period design, uniform model and multi-group comparison, etc. (Cheung, 2015; Al-Rahmi et al., 2022). In this study, the load factors were weighted to obtain the independent variables (personality traits), and through factor analysis and path analysis, the direct effect, indirect effect and total effect of the independent variables on the dependent variables (social sharing motivation) were obtained.

## Results

Table 3 reports the means, standard deviations, and correlations of the variables. Prior to testing our hypotheses, we first conducted a reliability analysis and validity analysis. The results showed an appropriate fitness between the model and data.

To analyse the reliability of the sample used in this survey, the valid data collected were tested to ascertain their reliability and validity. In this paper, reliability tests were first conducted using Cronbach’s α coefficient and composite reliability (CR). The test results showed that the Cronbach’s α coefficient and CR values of all variables were higher than 0.7 (as shown in Table 4), indicating that the measurement model exhibited a high degree of internal consistency and reliability. The validity tests were conducted mainly using convergent validity and discriminant validity. Convergent validity indicates whether the measurement terms of a variable are highly correlated with each other, and the main measures are factor loadings and average variance extracted (AVE). The test results show that all factor loadings were greater than the critical value of 0.7, and that the AVE values of all variables were greater than 0.5, thus indicating that the measurement model aggregated well. Discriminant validity indicates whether the correlation between the measurement terms of variables is as small as possible, which can be tested by reference to the relationship between the square root of the AVE value of each variable and the magnitude of the correlation coefficients among the variables. The test results showed that the square root of the AVE value for each variable was greater than the correlation coefficient between that variable and the other variables, thus indicating that the measurement model exhibited good discriminant validity.

**Table 4:** Reliability analysis indicators and results.

Variable	Measurement item	Standard factor loadings	Cronbach’s α	AVE value	CR value
Extraversion	EX1	0.801	0.893	0.686	0.897
	EX2	0.888			
	EX3	0.914			
	EX4	0.728			
Conscientiousness	CO 1	0.798	0.894	0.740	0.895
	CO 2	0.903			
	CO 3	0.879			
Neuroticism	NE 1	0.741	0.883	0.656	0.884
	NE 2	0.77			
	NE 3	0.856			
	NE 4	0.87			
Image Management	XX1	0.894	0.952	0.743	0.953
	XX2	0.916			
	XX3	0.929			
	XX4	0.902			
	XX5	0.796			
	XX6	0.795			
	XX7	0.784			
Altruism	LT1	0.794	0.839	0.642	0.842
	LT2	0.739			
	LT3	0.858			
Emotional Expression	QG1	0.911	0.932	0.774	0.932
	QG2	0.873			
	QG3	0.894			
	QG4	0.843			
Pastime and Conformity	XQ1	0.675	0.891	0.686	0.896
	XQ2	0.91			
	XQ3	0.916			
	XQ4	0.78			

According to Tables 5, 6, the KMO value is greater than 0.7, so the data are valid. The KMO test is used to check the correlation and partial correlation between variables, and its value ranges from 0 to 1. The closer the KMO statistic is to 1, the stronger the correlation between variables, the weaker the partial correlation, and the better the effect of factor analysis. In the actual analysis, when the KMO statistic is above 0.7, the effect is better. When the KMO statistic is below 0.5, it is not suitable to apply the factor analysis method. It is necessary to redesign the variable structure or adopt other statistical analysis methods. The commonality value of all research items is greater than 0.4, so there is no need to eliminate information, and all information can be effectively extracted. Commonality represents the amount of information that can be extracted from a certain item. The higher the commonality value is, the higher the index that can be explained by the principal components, and the more information that can be extracted. Generally, 0.4 is taken as the standard.

**Table 5:** Validity analysis indicators and results of personality trait scale.

Variable	Measurement item	Subject	Standard factor loadings			Commonality
			1	2	3	
Conscientiousness	CO1	-	−0.006	0.236	0.862	0.799
	CO2	-	0.013	0.376	0.833	0.836
	CO3	-	−0.032	0.382	0.822	0.822
Extraversion	EX1	-	0.04	0.612	0.576	0.709
	EX2	-	−0.005	0.82	0.395	0.829
	EX3	-	0.036	0.834	0.396	0.853
	EX4	-	0.117	0.823	0.222	0.74
Neuroticism	NE1	-	0.814	0.219	−0.014	0.711
	NE2	-	0.844	0.031	−0.065	0.718
	NE3	-	0.886	−0.026	0.051	0.789
	NE4	-	0.894	−0.047	0.03	0.802
-	-	Characteristic root value (before rotation)	4.855	2.962	0.79	-
-	-	Variance explanation rate % (before rotation)	44.139%	26.923%	7.183%	-
-	-	Cumulative variance explanation rate % (before rotation)	44.139%	71.062%	78.244%	-
-	-	Characteristic root value (after rotation)	2.977	2.815	2.814	-
-	-	Variance explanation rate % (after rotation)	27.068%	25.591%	25.585%	-
-	-	Cumulative variance explanation rate % (after rotation)	27.068%	52.659%	78.244%	-
-	-	KMO value	0.878			-
-	-	Bartlett’s test	4381.737			-
-	-	*df*	55			-
-	-	*p* Value	0			-

**Table 6:** Validity analysis indicators and results of sharing motivation scale.

Variable	Measurement item	Subject	Standard factor loadings						Commonality
			1	2	3	4	5	6	
Image Management	XX1	-	0.794	0.258	0.154	0.298	0.205	0.025	0.853
	XX2	-	0.844	0.239	0.229	0.205	0.196	−0.044	0.903
	XX3	-	0.829	0.261	0.231	0.242	0.172	0.002	0.897
	XX4	-	0.784	0.213	0.265	0.251	0.159	0.173	0.848
	XX5	-	0.616	0.337	0.117	0.282	0.223	0.402	0.798
	XX6	-	0.583	0.29	0.39	0.179	0.137	0.438	0.819
	XX7	-	0.521	0.188	0.533	0.245	0.246	0.27	0.784
Altruism	LT1	-	0.462	0.098	0.572	0.307	0.139	0.326	0.769
	LT2	-	0.141	0.139	0.859	0.171	0.127	0.009	0.823
	LT3	-	0.278	0.231	0.656	0.169	0.452	0.093	0.803
Emotional Expression	QG1	-	0.411	0.299	0.284	0.645	0.299	0.058	0.849
	QG 2	-	0.31	0.218	0.455	0.674	0.229	−0.022	0.858
	QG 3	-	0.321	0.391	0.216	0.688	0.264	0.135	0.864
	QG 4	-	0.325	0.391	0.193	0.657	0.2	0.235	0.822
Pastime and Conformity	XQ1	-	0.12	0.776	0.414	0.148	−0.063	−0.023	0.815
	XQ2	-	0.322	0.763	0.128	0.258	0.28	0.004	0.847
	XQ3	-	0.384	0.716	0.133	0.219	0.333	0.089	0.845
	XQ4	-	0.274	0.685	−0.003	0.355	0.173	0.283	0.780
-	-	Characteristic root value(before rotation)	13.689	1.376	1.242	0.769	0.626	0.538	-
-	-	Variance explanation rate % (before rotation)	62.221%	6.254%	5.646%	3.497%	2.844%	2.448%	-
-	-	Cumulative variance explanation rate % (before rotation)	62.221%	68.475%	74.120%	77.618%	80.462%	82.909%	-
-	-	Characteristic root value (after rotation)	5.283	3.551	3.42	3.166	1.965	0.854	-
-	-	Variance Explanation rate % (after rotation)	24.013%	16.142%	15.547%	14.390%	8.933%	3.883%	-
-	-	Cumulative variance Explanation rate % (After Rotation)	24.013%	40.155%	55.702%	70.093%	79.026%	82.909%	-
-	-	KMO value	0.963						-
-	-	Bartlett’s test	12964.045						-
-	-	df	231						-
-	-	*p*-value	0						-

In this paper, the research models were tested using SPSS 23.0 and AMOS 23.0 software, and the results are shown in Table 7. All the model fit indicators were better than the recommended values, indicating a good fit. SPSS is the first statistical software in the world to adopt a graphical menu-driven interface. Its most prominent feature is that the operation interface is very friendly and the output results are beautiful. It displays almost all functions in a unified and standardized interface. It uses Windows to display various functions of data management and analysis methods, and dialog boxes to display various functional options.

**Table 7:** Model fit indices.

Fit index	Recommendation value	Actual value
χ2 /df	<3	2.748
AGFI	>0.8	0.878
RMSEA	<0.08	0.073
IFI	>0.9	0.92
NNFI	>0.9	0.908
CFI	>0.9	0.92

*χ*^2^/df is the ratio of cardinal values to degrees of freedom, AGFI is the adjusted goodness-of-fit index, RMSEA is the root mean square of the error of approximation, IFI is the incremental fitness index, NNFI is the nonnormative fit index, and CFI is the comparative fit index.

Image management motivation is users’ desire to shape a rationalized self-image through short-video sharing to gain a sense of self-satisfaction and social identity. The specific characteristics of the online environment provide the possibility for individuals to escape from the shackles of their real identities and material conditions, and internet users tend to conceal unfavourable information and convey an image that is favourable to themselves. Simultaneously, although social media provides a channel for sharers to experience emotional catharsis, users can confide with other users *via* the network and thus obtain comfort and seek social support. According to Table 8, conscientiousness is not significantly correlated with users’ image management motivations or emotion expression motivations, but it is positively correlated with altruism motivations; therefore, Hypotheses H1-a, H1-b, and H1-c are rejected. This research on the effects of extroversion on social sharing motivation showed that extroversion as a personality trait has significant positive effects on image management, altruism, emotional expression, and pastime and conformity, especially with respect to the latter motivation. Hypotheses H2-a, H2-b, H2-c and H2-d are confirmed. Highly neurotic individuals tend to be highly motivated by image management, altruism, emotional expression, and pastime and conformity; accordingly, Hypotheses H3-a, H3-b, H3-c, and H3-d are confirmed.

**Table 8:** Test results.

*X*	-	*Y*	Nonstandard path coefficient	S.E.	*Z*	*p*	Marker coefficient
Conscientiousness	→	Image management	0.106	0.103	1.026	0.305	0.077(ns)
Conscientiousness	→	Altruism	0.204	0.094	2.160	0.031	0.171*
Conscientiousness	→	Emotional expression	0.113	0.101	1.120	0.123	0.084(ns)
Conscientiousness	→	Pastime and conformity	−0.180	0.075	−2.398	0.016	−0.180*
Extroversion	→	Image management	0.473	0.094	5.014	0.000	0.379***
Extroversion	→	Altruism	0.392	0.086	4.534	0.000	0.362***
Extroversion	→	Emotional expression	0.430	0.092	4.672	0.000	0.355***
Extroversion	→	Pastime and conformity	0.530	0.073	7.250	0.000	0.586***
Neuroticism	→	Image management	0.276	0.051	5.429	0.000	0.221***
Neuroticism	→	Altruism	0.212	0.046	4.580	0.000	0.196***
Neuroticism	→	Emotional expression	0.336	0.050	6.691	0.000	0.279***
Neuroticism	→	Pastime and conformity	0.288	0.039	7.312	0.000	0.319***

*p = 0.05, **p = 0.01, ***p = 0.001, ns indicates not significant.

## Discussion

### Theoretical implications

From the study, we concluded that individuals who exhibit high levels of conscientiousness are organized, disciplined, and responsible, and this personality trait reduces the likelihood of posting problematic content and the need to engage in online catharsis or emotional expression. In addition, conscientiousness is negatively related to users’ pastimes and conformity motivations, thus suggesting that highly conscientious individuals are able to effectively control their needs in virtual environments, especially by avoiding information-sharing behaviours that are aimed at killing time and following new trends. These findings are consistent with those reported by Lee et al. (2014).

The research proves that extroverted users are more likely to approach others and to participate by sharing more content when using short-video platforms; they also tend to use the internet to enhance their social skills while engaging in many offline social relationships. For extroverted users, online information sharing not only expands offline social contact but also provides a broader means of offline social contact. Since extroverts are social, outgoing, talkative and energetic, they tend to seek more social interaction both online and offline. This mode of social contact facilitated by online and offline interaction provides evidence for the Matthew effect of “the rich get richer.” This conclusion is in line with the findings of Ross et al. (2009) and Zywica and Danowski (2008). In addition, we found that users with high extroversion exhibit altruistic motivation and that users with altruistic perceptions tend to participate in social sharing in a spirit of reciprocity and altruism and freely make sacrifices of their personal time and energy to contribute their knowledge to help others solve problems. There is a significant correlation between extroversion and altruism, a finding that has not been reported by extant studies on the correlations of social sharing motivations.

In addition, our study suggests that users with high levels of neuroticism have a stronger need to engage in video sharing and prefer to express themselves *via* online platforms; this behaviour is the opposite of the anxious, repressed, and sensitive behaviours that they present offline, where they do not actively engage with others. Virtual interactions tend to compensate for the psychological distress experienced by individuals with high levels of neuroticism in the context of social exposure, so individuals with high levels of neuroticism tend to express themselves on the internet, attract the attention of others, communicate online through comments and achieve emotional connection, which can compensate for their deficiencies concerning real social interactions. This finding further confirms the social compensation effect of “the poor getting richer” in the context of the internet and is consistent with the findings of Mehdizadeh (2010) and Ross et al. (2009). Additionally, individuals with high levels of neuroticism have strong motivations for image management, altruism, emotional expression, and pastime and conformity, thus suggesting that such users have a strong need to belong and a desire to gain social acceptance.

### Practical implications

Our study provides valuable practical implications for users, short-video content providers, and short-video media. Regarding users, once information has been shared with others on short-video media, it is almost impossible for users to fully control the flow of information. Therefore, the privacy risks associated with the use of short-video media should be constantly communicated to users, especially those who prefer to share short videos. In addition, due to the uniqueness of the big data era, users should consciously think outside the box to avoid being trapped in an “information-isolated island” when sharing and receiving information.

Short video content providers can design and package video content for various sharing motivations and psychological needs of users to stimulate their sharing behaviours. For example, for users’ image management motivations, they can try to “tag” the content so that users can attribute the tagging information conveyed by the content to personal and group characteristics and then show the information to the public through social sharing to build a virtual image and seek social recognition; for users’ altruism motivations, content providers can emphasize its communication value in content design so that users with altruistic motivations realize that the content is useful to others and thus are stimulated to share.

Short video media should develop personalized functions and services for users with different personality traits to meet their motivation for sharing. For example, conscientious users will focus on the authenticity and traceability of the shared content when sharing short videos, so short-video media should make an official judgement on the authenticity of the video content and allow each video to be traced back to its source through a link; users with the trait of extroversion will participate in sharing more content when using short-video platforms, so a short-video medium can allow them to embed multiple types of expressions in the sharing process, such as text, pictures, audio, etc., to effectively stimulate their interest in sharing.

### Limitations and future directions

The current study has limitations in certain aspects, which will inform and guide future research. First, the current study explored the relationships between user personality traits (conscientiousness, extraversion, and neuroticism) and short-video sharing motivations (image management, altruism, emotional expression, and pastime and conformity). The relationship between the Big Five personality traits of openness and agreeableness and short-video sharing motivation was not examined. Additionally, the current study did not address other short-video sharing motivations, such as information-seeking and convenience. Future research will examine the intrinsic link between a wider variety of personality traits and a more comprehensive range of sharing motivations.

Second, the group in this research was young. Nearly 80 % of the people were under 30 years old, and these people may be more inclined to interact and express themselves on short-video media. In the future, a larger number of samples should be used to provide full coverage of all age groups.

Third, because the participants were recruited from the WeChat platform, the sample may also be biased towards those who enjoy social interaction on the internet and sharing on short-video media. Traditional offline recruitment methods could be used in future research.

Fourth, because all of the participants were Chinese, there may be significant differences when the study is applied to other countries. In future studies, we will expand the scope of participants to other countries in Asia and the globe.

## Conclusion

Overall, our findings suggest that users’ personality traits influence their motivations to share and that individuals’ motivations to share short videos vary depending on their personality traits. Highly responsible users tend to share short videos for altruistic reasons rather than to fulfil their image management and emotional expression needs, and such users strictly manage their own online time, which is negatively related to their pastime and conformity motivations. In contrast, extraversion and neuroticism had a very significant positive effect on image management, altruism, emotional expression and pastime and conformity. This finding, which suggests that personalities that feature extraversion and neuroticism can be predictors of users’ sharing motivations, should therefore be considered in the context of both short-video content creation and short-video social platform construction with regard to factors related to users’ intrinsic personality traits. In addition, our study confirms not only the Matthew effect of “the rich getting richer” in the context of short-video social media but also the social compensation effect of “the poor getting richer,” which provides a more adequate theoretical basis and additional empirical evidence for future research on users’ sharing behaviours.

## Data availability statement

The original contributions presented in the study are included in the article/supplementary material, further inquiries can be directed to the corresponding author.

## Ethics statement

Ethical review and approval was not required for the study on human participants in accordance with the local legislation and institutional requirements. The patients/participants provided their written informed consent to participate in this study.

## Author contributions

ZD-Y conceived and designed the experiments and wrote the main manuscript. SZ performed the numerical analysis. All authors contributed to the article and approved the submitted version.

## Funding

This study received funding from State Key Laboratory of Communication Content Cognition, Phase I (Project No. A12001) and Heilongjiang Returnee Science Foundation (Project No. LC2018031).

## Conflict of interest

ZD-Y was employed by People’s Daily Online.

The remaining author declares that the research was conducted in the absence of any commercial or financial relationships that could be construed as a potential conflict of interest.

## Publisher’s note

All claims expressed in this article are solely those of the authors and do not necessarily represent those of their affiliated organizations, or those of the publisher, the editors and the reviewers. Any product that may be evaluated in this article, or claim that may be made by its manufacturer, is not guaranteed or endorsed by the publisher.
